# The prognostic factors of Ewing sarcoma/peripheral primitive neuroectodermal tumor: A retrospective analysis of 67 patients at a single center

**DOI:** 10.1097/MD.0000000000029564

**Published:** 2022-07-22

**Authors:** Jing Peng, Xing Min Han

**Affiliations:** a Department of Nuclear Medicine, the First Affiliated Hospital of Zhengzhou University, Henan Medical Key Laboratory of Molecular Imaging, Zhengzhou City, Henan Province, China.

**Keywords:** Ewing sarcoma, influencing factor, peripheral primitive neuroectodermal neoplasms, primitive neuroectodermal tumor, prognosis, survival, therapy

## Abstract

To investigate the characteristics and factors that impact the prognosis of Ewing sarcoma/peripheral primitive neuroectodermal tumor (ES/pPNET) patients. We retrospectively analyzed ES/pPNET patients diagnosed at our hospital from January 2011 to December 2020. We used 1-way analysis of variance to investigate whether the age differences between different subgroups were statistically significant and used the Kaplan–Meier method and Cox regression model for the survival analysis. Of the 67 included patients, 13 had central nervous system PNET, and 54 had ES/pPNET. The median survival time of the 54 ES/pPNET patients was 18 months; the 1-year, 3-year and 5-year progression-free survival rates were 37.0% and 9.3% and 1.9%, respectively; and the 1-year, 3-year and 5-year overall survival (OS) rates were 66.7%, 27.8%, and 7.4%, respectively. The 1-way analysis of variance results showed no statistically significant age difference between the different subgroups (*P* = .127 between the central nervous system PNET and ES/pPNET groups, *P* = .764 between different subgroups within the ES/pPNET group). The univariate survival analysis showed that metastasis at diagnosis and the treatment method were independent factors affecting the OS rate of ES/pPNET patient (*P* = .003 and 0.000, respectively). The multivariate survival analysis also showed that the treatment method and metastasis at diagnosis were related factors affecting OS (*P* = .025 and 0.001, respectively). The prognosis of patients with primitive neuroectodermal tumors is poor. The treatment method and metastasis at the time of diagnosis influences ES/pPNET patient survival time, but there is no significant tumor site-dependent correlation with patient age or sex.

## 1. Introduction

Peripheral primitive neuroectodermal tumors and Ewing sarcoma belong to the Ewing sarcoma family. The 2016 tumor classification categorizes these tumors as mesenchymal tumors referred to as Ewing sarcoma/peripheral primitive neuroectodermal tumors (ES/pPNET).^[1]^ ES/pPNET is rare, accounting for <1% of all sarcomas. Relevant studies have revealed that the clinical and imaging manifestations of ES/pPNET are not typical, and the disease has a high degree of malignancy and a rapid progression. Most patients have metastasis at diagnosis, and the 2-year survival rate is only approximately 30%.^[2,3]^ Moreover, a literature review revealed that most ES/pPNET-related publications are case reports.^[4,5]^ There are few studies on the disease characteristics and patient survival prognoses.

Therefore, it is urgent to understand the prognostic impact of tumor occurrence, development, characteristics, and progression rate along with the patient survival rate and treatment mode. Data on the pathologically confirmed ES/pPNET cases in our hospital from January 2011 to December 2020 were retrospectively collected, and the clinical and imaging data of the patients were analyzed. The characteristics and prognostic factors of ES/pPNET revealed in this study are expected to be helpful to clinicians.

## 2. Patients and Methods

### 2.1. Clinical data

Patients diagnosed with primitive neuroectodermal tumors at our hospital from January 2011 to December 2020 were enrolled. The inclusion criteria were as follows: the tumor was confirmed by pathology in our hospital as a primordial neuroectodermal tumor of the ES/pPNET type; complete medical records available, such as records of medical visits and imaging examinations, were available; and follow-up was possible. The exclusion criteria were as follows: incomplete clinical data of the patient, for example, the patient underwent a pathological examination and partial treatment in another hospital, and the data could not be obtained; patients who were complicated with other fatal malignant diseases; and patients who were lost to follow-up.

### 2.2. Methods

The clinical data of the enrolled patients were retrospectively analyzed, the prognostic information was followed up, and the data were subsequently analyzed by statistical software. The main statistical data included sex, age, site of onset, the maximum diameter of the tumor, the treatment method, metastasis at diagnosis, disease progression, and patient survival during follow-up. Progression-free survival (PFS) and overall survival (OS) times were calculated. The follow-up time was calculated from the date of diagnosis to the date of the occurrence of outcome events or the date of the final follow-up. The follow-up method involved consulting the medical and imaging data, performing a telephone follow-up and recording patient treatment responses and survival outcomes.

Due to the small number of central primitive neuroectodermal tumor (cPNET) patients enrolled, we mainly analyzed disease progression and prognostic factors in the 54 ES/pPNET patients. The incidence of cPNET and disease progression in the 13 patients were briefly analyzed. In addition, cPNET patients were also included in the analysis of age differences among patient groups with different onset sites and the determination of the impact of the onset site on patient survival.

The study protocol was approved by the ethics review board of the First Affiliated Hospital of Zhengzhou University. We obtained oral informed consent from all study participants. All of the procedures were performed in accordance with the Declaration of Helsinki and relevant policies in China.

### 2.3. Statistical analysis

SPSS 25.0 software was used for the statistical analysis. One-way analysis of variance (ANOVA) was used to determine whether age differences were statistically significant among patients with different sites of onset. The Kaplan–Meier method was used to calculate the PFS and OS rates at 1, 3, and 5 years and to analyze the prognostic effects of sex, age, site of onset, treatment method, and metastasis at diagnosis. Log-rank tests were used to determine statistical significance. A Cox risk regression model was used for the multivariate survival analysis to determine the prognostic factors. A *P* value of <.05 was considered statistically significant.

## 3. Results

### 3.1. Patient characteristics and prognoses

A total of 67 patients were enrolled. There were 13 patients with cPNET, with an average age of 16.5 ± 11 years (1.5–38 years). Of those with cPNET, 8 were male and 5 were female; 6 had tumors located in the spinal canal, and 7 had tumors located in the brain. There were 54 patients with ES/pPNET (40 with soft tissue tumors, 3 with bone tumors, and 11 with visceral tumors), with an average age of 22.6 ± 15.9 years (1–59 years). Of those with ES/pPNET, 26 were male and 28 were female; and 8 had tumors located in nonmidline areas (e.g., the shoulders and limbs), and 59 had tumors located in midline areas (e.g., the head and face, chest and abdomen).

During data analysis, it was found that the incidence of primitive neuroectodermal tumor has increased in recent years, and most patients experience relapse and death within a short period after treatment. The maximum PFS time of all 67 patients was 73 months, the minimum was 0.5 months, and the median was 9 months. The 1-year, 3-year, and 5-year PFS rates were 38.8%, 7.5%, and 1.5%, respectively. The longest OS duration was 106 months, the shortest was 1 month, and the median was 18 months. The 1-year, 3-year, and 5-year OS rates were 67.2%, 26.8%, and 9.0%, respectively.

Among the 13 cPNET patients, 1 (7.7%) received surgery alone, 1 received (7.7%) chemotherapy alone, and 11 (84.6%) received surgery combined with radiotherapy and chemotherapy. The longest PFS duration was 20 months, the shortest was 2 months, and the median was 10 months. The PFS rates of the 13 cases at 1 year, 3 years, and 5 years were 46.2%, 0%, and 0%, respectively. The longest OS duration was 106 months, the shortest was 7 months, and the median was 15 months. The OS rates at 1 year, 3 years, and 5 years were 69.2%, 23.1%, and 1.5%, respectively.

Among the 54 ES/pPNET patients, 2 patients (3.7%) had no treatment, 11 patients (20.4%) had surgery only, 4 patients (7.4%) had chemotherapy only, and 37 patients (68.5%) had surgery combined with radiotherapy and chemotherapy. There were 26 patients (48.1%) with metastasis at diagnosis, of which 20 (76.9%) had regional lymph node metastases. The longest PFS duration was 73 months, the shortest was 0.5 months, and the median was 9 months. The 1-year, 3-year, and 5-year PFS rates were 37.0%, 9.3%, and 1.9%, respectively. The longest OS duration was 96 months, the shortest was 1 month, and the median was 18 months. The OS rates at 1 year, 3 years, and 5 years were 66.7%, 27.8%, and 7.4%, respectively.

### 3.2. One-way ANOVA: The age difference among groups with different disease sites

We divided the patients into the cPNET and the ES/pPNET groups. The 54 ES/pPNET patients were further divided into groups depending on whether their disease site was soft tissue, bone, or a visceral organ. Then, the age differences among the related groups were studied by 1-way ANOVA.

The 1-way ANOVA results showed that there was no significant difference in age based on sites of onset. The comparison of the cPNET group with the ES/pPNET group showed *F* = 2.386, *P* = .127 > .05. Among the ES/pPNET patients, there was no statistical significance in the ages of the patients in the soft tissue group, the viscera group and the bone group, with a statistical result of *F* = 0.270, *P* = .764 > .05.

### 3.3. Kaplan–Meier analysis: The influence of location on the OS outcomes of ES/pPNET patients

cPNET patients were included in the analysis. After the cPNET and ES/pPNET groups were analyzed, the patients were further divided into the midline and nonmidline groups for analysis. The Kaplan–Meier analysis showed that the OS outcomes between the ES/pPNET group and the cPNET group were not significantly different (χ^2^ = 0.988, *P* = .320 > 0.05). There was also no significant difference in OS outcomes between the midline and nonmidline groups (χ^2^ = 1.891, *P* = .169 > 0.05). In addition, among the 54 ES/pPNET patients, there was no significant difference in OS outcomes among the soft tissue group, viscera group, and bone group (χ^2^ = 5.339, *P* = .069 > 0.05)

### 3.4. Kaplan–Meier analysis: Prognostic factor analysis of the 54 ES/pPNET patients

**Figure 1.**
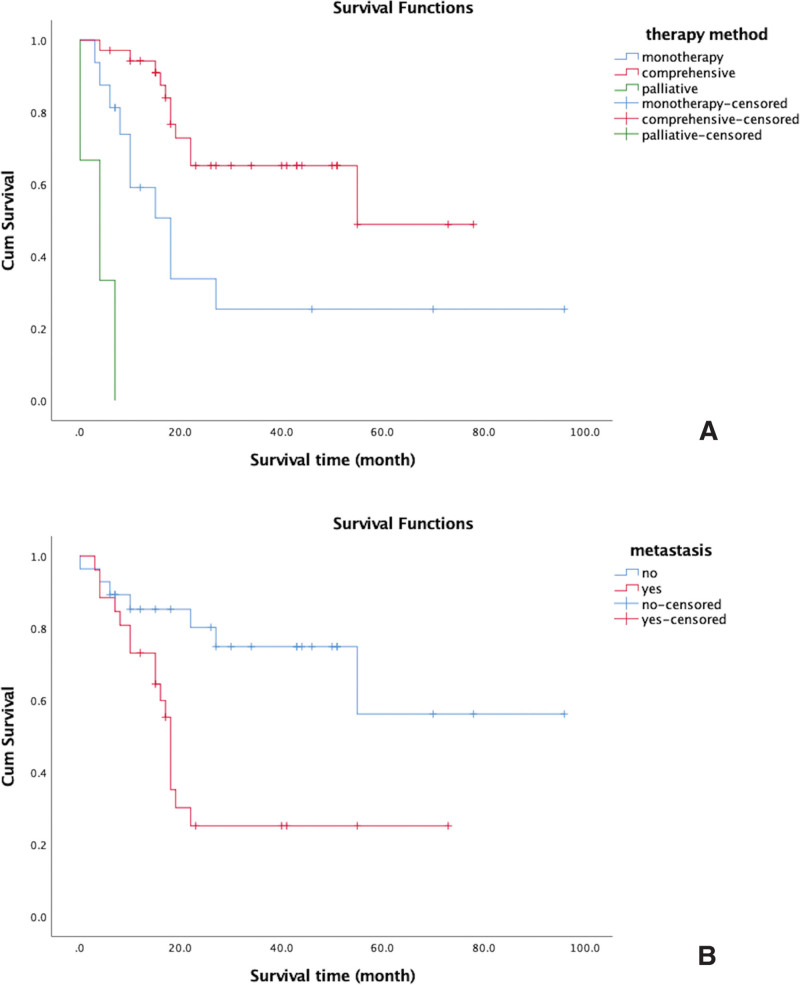
Kaplan–Meier analysis of the effect of treatment and metastasis on overall survival. (A) Comparison between surgery combined with chemoradiotherapy, surgery alone, or chemotherapy and palliative care. The red line is surgery combined with chemoradiotherapy, the blue line is surgery or chemotherapy alone, and the green line is palliative care. As shown in the figure, there was a significant difference in OS among patients treated with the 3 types of treatment. Patients treated with surgery combined with chemoradiotherapy had a longer OS than those treated with surgery or chemotherapy alone, and patients treated with surgery or chemotherapy alone had a longer OS than those treated with palliative care alone. (B) Comparison between patients with and without metastasis. The blue line indicates no metastasis, and the red line indicates metastasis. The blue line indicates no transfer, and the red line indicates a transfer. The mean duration of OS was shorter in patients with metastasis at diagnosis than in patients without metastasis. OS = overall survival.

Kaplan–Meier survival analysis showed that treatment method and metastasis at diagnosis were independent factors affecting OS outcomes, while sex, age, tumor size, and tumor location were not associated with OS outcomes, as shown in Table 1. Among the different treatment modalities, the OS duration of patients treated with surgery combined with radiotherapy and chemotherapy was longer than that of patients treated with surgery or chemotherapy alone; additionally, the OS duration of patients treated with surgery or chemotherapy alone was longer than that of patients who received only palliative care (χ^2^ = 38.628, *P* = .000), as shown in Figure 1A. The OS duration of the 3 patients who received palliative care ranged from <0.5 to 7 months. The mean OS duration of patients with metastases at diagnosis was shorter than that of patients without metastases (χ^2^ = 8.839, *P* = .003), as shown in Figure 1B. Of the 26 patients with metastatic disease, 76.9% had regional lymph node metastasis.

**Table 1:** Univariate survival analysis of 54 ES/pPNET patients.

Classification		Total	Number of deaths	χ^2^	*P* value
Location	Midline	46	23	1.891	.169
	Nonmidline	8	1		
Histological location	Soft tissue	40	16	5.339	.069
	Organs	11	8		
	Bone	3	0		
Classification	cPNET	13	8	0.988	.320
	ES/pPNET	54	24		
Therapies	Only S or C	16	10	38.628	.000
	S, R, and C	35	11		
	P	3	3		
Sex	Male	26	12	0.222	.637
	Female	28	12		
Metastasis or not	Metastasis	26	17	8.839	.003
	No metastasis	28	7		
Age	≥20	25	10	0.119	.730
	<20	29	14		

C = chemotherapy, cPNET = central nervous system primitive neuroectodermal tumor, ES/pPNET = Ewing’s sarcoma/peripheral primitive neuroectodermal tumor, P = palliative care, R = radiotherapy, S = surgery.

### 3.5. Multivariate survival analysis: Factors influencing the OS outcomes of ES/pPNET patients

The multivariate survival analysis showed that metastasis and treatment mode were related to ES/pPNET patient OS outcomes, as shown in Table 2.

**Table 2:** Multivariate survival analysis of 54 ES/pPNET patients.

Variate	Regression coefficient	Standard error	Statistic	*P* value	Hazard ratio	95% CI	
						Lower	Upper
Metastasis	1.138	0.503	5.026	.025	3.120	1.154	8.437
Therapy	–1.675	0.526	10.140	.001	0.187	0.067	0.525

CI = confidence interval, ES/pPNET = Ewing’s sarcoma/peripheral primitive neuroectodermal tumor.

## 4. Discussion

In this study, we found that ES/pPNET is more common than cPNET, the age of onset is during adolescence, and the site of onset is the abdomen and pelvic soft tissue. The survival rate of patients is low. In this study, the median PFS times of ES/pPNET and cPNET patients were 9 months and 10 months, respectively, and the median OS times were was 18 months and 15 months, respectively. The treatment mode and stage were independent factors affecting survival time. There was no significant correlation between sex, age, or disease site and survival time.

According to the literature, the first identified primitive neuroectodermal tumor was diagnosed by Hart and Earle^[6]^ in 1973 and occurred in the brain. Primitive neuroectodermal neoplasms are rare, originating from primitive neuroepithelial cells and mainly composed of undifferentiated small round tumor cells.^[7]^ Clinically, primitive neuroectodermal tumors are often divided into cPNET and pPNET; however, molecular genetic studies have shown that both pPNET and ES originate from neural crest embryonic cells and have the same T (11, 22)(q24, q12) chromosomal heterotopic status; thus, these mesenchymal tumors are classified as ES/pPNET.^[1]^

### 4.1. General clinical features

In this study, 13 of the 67 patients had cPNET (19.4%), with an average age of 16.5 ± 11 years (1.5–38 years). cPNET were located in the spinal canals of 6 patients, the brains of 7 patients, and the supratentorial areas of 6 patients. These findings were consistent with the morbidity characteristics reported in the literature. ES/pPNET originate from primitive neuroepithelial cells and have the potential to differentiate along multiple lineages; thus, ES/pPNET can occur in all parts of the body.^[8]^ cPNET are rarer than ES/pPNET and are more common in children aged <10 years. The most common sites are the brain and spinal cord. Those occurring in the parenchyma of the brain are mostly supratentorial, such as the frontal-parietal lobe.^[9]^

The average age of the 54 ES/pPNET patients in this study was 22.6 ± 15.9 years (1–59 years). There were 40 cases of soft tissue tumors, 3 cases of bone tumors, and 11 cases of visceral tumors. Askin tumors were found in 5 of the 8 patients with primary thoracic tumors. Gao et al^[10]^ reported that ES/pPNET are most frequently located in the soft tissue of the abdomen and pelvis, followed by the thorax and lung, whereas ES/pPNET in the chest wall, also known as Askin tumors, are rare. Therefore, after review of the previous literature, we believe that ES/pPNET is most likely to occur in the soft tissue throughout the body, while bone and internal organs are the next most common sites.^[5,11]^ The age of onset varies slightly from study to study. The median age of the 14 ES/pPNET patients described by Sun Pengtao et al was 34 years.^[12]^ Tan et al^[13]^ reported a median age of 30 years and noted that the mean age of patients with primary lesions in the bone was lower than that of patients with primary lesions in the soft tissue and viscera.

However, in our study, the 1-way ANOVA results showed no statistically significant difference in the age of the patients based on tumor site. The results of the comparison between the cPNET group and ES/pPNET group were *F* = 2.386, *P* = .127. The comparison between the ES/pPNET patient soft tissue, visceral, and bone groups was *F* = 0.270, *P* = .764. This result is different from that reported by Tan et al.^[13]^ In addition, regarding the effect of sex, studies have revealed that ES/pPNET is more common in women.^[14]^ In this study, of the 54 ES/pPNET patients, 26 were males and 28 were females, and there was no significant sex difference in the incidence.

We believe that these contradictory results are due to population differences across studies. As all of the studies involve small cohorts, the interpretation of results may have limitations. Therefore, we expect a comprehensive analysis of multicenter, large-sample study data to resolve the contradictions among the current studies. However, the disease is generally more common in adolescents, and peripheral soft tissue tumors are the most common site of onset.

### 4.2. Survival outcomes and prognoses of ES/pPNET patients

The occurrence and development of ES/pPNET have the following characteristics: first, a fast growth rate leads to large tumors at diagnosis, especially when the tumor arises in the abdominal and pelvic organs and soft tissues. Second, high invasiveness and frequently metastasized at diagnosis. Third, poor patient prognosis due to low detection rates and late diagnoses. ES/pPNET are prone to relapse in the short term after treatment, and the survival prognosis of patients is poor. Fourth, there are no standard treatment guidelines for the disease.

The incidence of primitive neuroectodermal tumors is low, but the survival rate is not optimistic.

In this study, the median PFS duration for the 13 cPNET patients was 10 months, and the median OS duration was 15 months. The 1-year, 3-year, and 5-year PFS rates were 46.2%, 0%, and 0%, respectively, and the 1-year, 3-year, and 5-year OS rates were 69.2%, 23.1%, and 1.5%, respectively. Among the 54 ES/pPNET patients, the median PFS duration was 9 months, and the median OS duration was 18 months. The 1-year, 3-year, and 5-year PFS rates were 37.0%, 9.3%, and 1.9%, respectively. The OS rates at 1 year, 3 years, and 5 years were 66.7%, 27.8%, and 7.4%, respectively.

A 2018 study reported 99 patients with 1-year, 3-year, and 5-year OS rates of 79.2%, 63.9%, and 56.1%, respectively, and PFS rates of 42.7%, 25.7%, and 19.8, respectively.^[15]^ Other studies on ES/pPNET have been conducted. Lan et al^[16]^ analyzed the prognosis of 47 ES/pPNET patients and found that the median OS duration was 23.5 months, and the 5-year survival rate was only 33.8%. Due to the low incidence of cPNET, few studies with large samples have been conducted to analyze patient prognosis independently. A 2014 single-center study of 9 cPNET patients over 10 years showed a 2-year survival rate of only 20%.^[17]^

In conclusion, combining previous literature reports and the results of this study indicates that primitive neuroectodermal tumors develop quickly and have a poor prognosis. Therefore, it is necessary to understand the prognostic factors further to help predict disease development and take timely targeted treatment measures in clinical diagnosis and treatment processes.

### 4.3. Influencing factors of ES/pPNET patient OS outcomes

Data on 54 patients with ES/pPNET were statistically analyzed, and the Kaplan–Meier survival analysis showed that treatment method and metastasis at diagnosis were independent factors affecting OS outcomes. There was no significant correlation between sex, age, or disease location and the OS rate. The Cox multivariate survival analysis also suggested that treatment method and metastasis at diagnosis were prognostic factors. The prognosis of patients treated with surgery combined with radiotherapy and chemotherapy was significantly better than that of patients treated with surgery or chemotherapy alone (χ^2^ = 38.628, *P* = .000). The prognosis of patients with metastatic disease was significantly worse than that of patients without metastasis (χ^2^ = 8.839, *P* = .003).

Regarding the factors influencing the OS outcomes of ES/pPNET patients, Kong Lingfei et al’s^[18]^ study on 15 patients showed that age, tumor location, and tumor size were all factors influencing the prognosis of ES/pPNET patients. Those <14 years of age with nonmidline tumors and small tumors and who underwent complete resection had a better prognosis.^[18]^ However, some studies have shown that stage and treatment mode affect prognosis, while age and sex are unrelated.^[19]^

In contrast to the results of Kong Lingfei et al’s^[18]^ study, there was no statistically significant difference in OS rates between patients with tumors at midline and nonmidline sites among the 54 cases (χ^2^ = 1.891, *P* = .169). We hypothesize that this is due to the low number of patients with nonmidline tumors. In this study, 7 patients had nonmidline tumors and 47 had midline tumors. There was no significant difference in the OS rate among patients with soft tissue, visceral, and bone tumors (χ^2^ = 5.339, *P* = .069). In addition, according to the study and analysis by Zhang Zongyin et al^[3]^ in 2019, the OS duration of those under 20 years old was longer than that of those over 20 years old (*P* = .007). In this study, there were 25 patients over 20 years of age and 29 patients under 20 years of age, and there was no significant difference in OS outcomes between the 2 groups (χ^2^ = 0.119, *P* = .730).

We also reviewed some large sample size studies. For example, Gao et al^[10]^ summarized 89 ES/pPNET patients in 2019, and Jiang et al^[20^ performed a database analysis of over 3000 patients in 2018. Combined with the results of this study, it is concluded that the tumor stage (metastasis) and treatment mode are critical prognostic factors.

In conclusion, primitive neuroectodermal neoplasms tend to occur in the soft tissues of the abdomen and pelvis of adolescents, with rapid progression and poor prognosis. Metastasis at diagnosis and treatment method are the key factors affecting patient prognosis. Early diagnosis of primary and metastatic lesions is critical for improving prognosis, and surgical resection combined with radiotherapy and chemotherapy should be initiated as early as possible.

### 4.4. Limitations of this study

First, the number of cases in this study is still relatively small. Second, this study is a retrospective study covering a long time period, and patient prognosis may have been affected by factors such as clinician experience and patient compliance. We expect that large-sample-size prospective studies will provide more detailed and accurate clinical conclusions in the future.

## Acknowledgments

We thank Dr XV Shasha for the statistical consultation. In addition, we thank the American Journal Specialist team for the language editing and polishing of this manuscript.

## Author contributions

Xing Min Han is mainly responsible for guiding the research direction and content, and reviewing the first draft of the article. Jing Peng was mainly responsible for case collection, data analysis, and draft writing.
